# Lymphoma in Sub-Saharan Africa: a scoping review of the epidemiology, treatment challenges, and patient pathways

**DOI:** 10.1007/s10552-024-01922-z

**Published:** 2024-10-17

**Authors:** Obsie T. Baissa, Tomer Ben-Shushan, Ora Paltiel

**Affiliations:** 1 Braun School of Public Health and Community Medicine, Hadassah Medical Organization, Faculty of Medicine, Hebrew University of Jerusalem, Jerusalem, Israel; 2 The Berman Medical Library, Hebrew University of Jerusalem, Jerusalem, Israel

**Keywords:** Lymphoma, NHL, HL, Scoping review, Sub-Saharan Africa, Patient pathways, Epidemiology

## Abstract

**Purpose:**

Improving cancer outcomes in Sub-Saharan Africa (SSA) requires effective implementation of evidence-based strategies. This scoping review maps the evidence on lymphoma epidemiology, treatment challenges, and patient pathways in SSA from 2011 to 2022.

**Methods:**

A comprehensive three-step search was conducted without language restrictions.

**Results:**

Eighty-four publications were included, 83% published after 2017. Southern and Eastern Africa led in output. Most studies were chart reviews (47.6%) and cohort studies (25%). NHL accounted for over 80% of cases, with an age-standardized rate (ASR) reaching 10.9/100,000, while HL had an ASR of 0.4–2.3/100,000. Compared to studies in Europe and US, SSA studies reported lower incidence rates, higher HIV comorbidity, and younger median ages. Diagnosis is often delayed, incomplete and lacks sub-classification with HIV and tuberculosis further complicating care. One-year survival rates are around 50% for NHL and over 75% for HL. Treatment is well-tolerated with an acceptable treatment-related mortality rate. However, outcomes are affected by diagnostic delays, late presentations, and treatment abandonment. Non-clinical aspects of care such as financial constraints negatively impact patient pathways.

**Conclusion:**

Addressing diagnostic delays, misdiagnosis, and treatment abandonment is crucial. Strengthening care access, diagnostics, and integrating innovative strategies including a multidisciplinary approach and re-designing efficient clinical diagnostic pathways are vital.

**Supplementary Information:**

The online version contains supplementary material available at 10.1007/s10552-024-01922-z.

## Introduction

Cancer is increasingly becoming a public health challenge in Africa [1]. According to the 2020 International agency for cancer research (IARC) estimate, Africa has a cancer incidence rate of 132.1 per 100,000 population [2]. However, this figure is projected to double by 2040. [2] Furthermore, the ratio of cancer mortality in Africa when compared to its incidence rate (88.8 vs. 132.1. per 100,000, respectively) [1] is significantly higher than in other regions. Cancer continues to be undiagnosed, unreported, untreated, and un-palliated in Sub-Saharan Africa (SSA) [3].

Evidence-based strategies and policies are essential in the control and prevention of cancer [1, 4]. Availability of data on cancer incidence and mortality in the SSA is limited [5]. In 2008, cancer registries in Africa covered only 1% of its population reflecting a significant gap in data availability regarding cancer incidence and mortality. [6] Over the last decade, progress has been made in expanding cancer registries, with coverage now reaching 11% of the population [6]. Since its inception in 2012, the African Cancer Registry Network (AFCRN) established 31 population-based cancer registries in 22 SSA countries, with the aim of availing more data and assessing the cancer epidemiology in the region [6]. While this is an advance in the right direction, further expansion and refinement of these registries are vital for enhancing our understanding of cancer epidemiology in the region [6].

Lymphomas contribute to over half of all hematologic cancers in the SSA region and in 2020, contributed to 10% of all cancer-related deaths in the region [7]. NHL was the sixth most common cancer in SSA with an estimated incidence of over 50,000 cases per year. On the other hand, Hodgkins’s lymphoma has an estimated incidence of 10,300 cases per year [7]. Although lymphomas are highly treatable, and significant progress has been made in the cure of these malignancies globally, mortality from lymphomas are unacceptably high in SSA. According to GLOBOCON report, there were estimated 30,000 and 4,300 deaths per year among NHL and HL patients, respectively [3, 7]. Therefore, understanding the epidemiology, diagnostic process and treatment outcomes of these cancers in SSA is vital.

This scoping review addresses the growing, but still incomplete and evolving body of knowledge concerning lymphoma, including the epidemiology, diagnosis, treatment, treatment pathways and prognosis in SSA. The main objective of this paper is to map the overall lymphoma landscape in SSA and address research gaps and advice on policy.

## Methods

The primary aim of this scoping review is to map the existing literature on the epidemiology, clinical characteristics, and therapeutic challenges and pathways of lymphoma patients in SSA from 2011 to 2022, using standardized protocols as well as exhaustive and reproducible methodological tools, in order to inform evidence-based cancer control and cancer care strategies in the region. The themes examined including study location, sample size, study design, study theme and main findings. A preliminary search in the Cochrane and Medline database did not show any ongoing scoping review on the topic. This review was conducted in accordance with the JBI methodology for scoping reviews and PRISMA-ScR Checklist [8].

### Eligibility criteria

We included peer reviewed articles from the SSA region which were published between 2011 and December 2022. Studies which primarily focused on lymphoproliferative diseases such as adult Hodgkin lymphoma (HL) and non-Hodgkin lymphoma (NHL) above the age of 16 or mixed studies with adult and pediatric populations were included. There was no language restriction in the search and we included all studies with a sample size of at least 15 or more participants. For articles with double publications, the most recent and comprehensive publication was used.

We excluded studies conducted among populations of SSA origin but residing outside of SSA. We also excluded studies focusing only on cutaneous lymphoma. Studies that used radiologic diagnostic criteria were also excluded. However, population-based registries were included even if classification criteria were not based solely on histology.

This review included both observational and interventional studies including those with descriptive and analytical study designs such as case control, cohort and cross-sectional studies. Additionally, interventional studies were also included in the analysis. However, case reports, conference proceedings, and narrative reviews were excluded.

### Search strategy

An initial limited search of PubMed (Medline) and Embase databases was conducted followed by an analysis of text words contained in the title and abstract, and of the index terms used to describe the article. A second search using all identified keywords and index terms was then undertaken across PubMed, Embase, and Web of Science databases. Thirdly, the reference lists of all identified reports and articles were searched manually for additional studies. A librarian was involved in designing and conducting of the search strategy. There was no language restriction in the search strategy. Database specific broad search terms such as lymphoma, hematologic malignancy, lymphoid malignancies, Africa, Africa south of Sahara and individual countries were included in the search strategy. Please see Appendix 1 for details of the search strategy.

All identified citations were uploaded in to Rayyan software for screening and extraction [9]. The two authors (OP and OB) conducted a review independently on the articles. Any disagreements that arose during the selection process were resolved through discussions. By design, risk of bias assessment is not be required for the scoping review, hence no article was excluded due to quality assessment.

### Data extraction

A data extraction form was developed by the team and were tested before use to ensure all relevant data would be extracted. Study characteristics including country, design, study population, and key findings were extracted. The two authors (OP and OB) conducted the data extractions. As shown in Table 1, a logical and descriptive summary of the results per research question was presented via a charting table to map the existing literature in alignment with the objectives. For the result section, we have synthesized the core themes and summarized the studies in seven sub-sections including incidence, clinico-pathologic features, risk factors, diagnostics, treatment and treatment outcomes, patient pathways and finally care models.

**Table 1:** Data charting form in reverse chronological order (Most recent first): lymphoma in Sub-Saharan Africa: A scoping review

First author (year) country	Study design	Participants	Theme	Summary of findings
Degu Kenya [10]	Hospital-based prospective cohort	50 lymphoma patients 68% had stage 3 or 4 disease	Treatment outcome	Mortality rate at 12 months was 10% Disease progression was reported in 20% of the patients Partial remission in 42% of patients only 2% of patients had complete remission
Gondwe Malawi [11]	Cohort	412 lymphoma patients both in pre universal and post universal ART policy 50% HIV prevalence	Epidemiology/HIV	Pre-ART lymphoma subtypes distribution—DLBCL (48%), low-grade NHL (12%), HL (11%) Post-universal ART lymphoma subtypes distribution—DLBCL (43%), low-grade NHL (11%), &HL (8%) No significant changes in incident lymphoma subtypes after implementation of universal ART, but HIV was better controlled
Ruffieux South Africa [12]	Cohort	1060 cases of HIV associated NHL Median age – 36 Median CD count—200	Epidemiology	Lower CD count associated with increased incidence of NHL
Ugwu Nigeria [13]	Chart review	135 cases of hematologic malignancies Median age—49	Epidemiology	NHL was the most common hematologic cancer accounting for 25.9% followed by CLL (24.4%), and HL (10.4%)
Parkin Zimbabwe [14]	Cancer registry	Comparison of the 1963–72 VS 2011–2015 cancer registries in Bulawayo	Epidemiology	Incidence of NHL increased by 6.3 times in males and 17.8 times in females in the 2011–2015 period compared to 1963–72
Cai SSA [15]	Population- based cancer registry analysis	Global burden of disease study	Epidemiology	The trends in SSA did not show significant change in both incidence and mortality Negative trends in incidence were observed in Zimbabwe, Ethiopia, Malawi, South Sudan and Central Africa Republic Overall ASR in SSA was below 2.5
*Kimani Malawi [16]	Single arm interventional study	37 patients with DLBCL 73% (27) were HIV+ ECOG 0–2 performance status 54% had stage 3 or 4 disease	Treatment outcome	OS and PFS among DLBCL patients treated with R-CHOP was 68% and 59% respectively at 12 months No difference on OS &PFS based on HIV status 11% treatment-related mortality HIV + DLBCL patients had lower treatment intensity and frequency due to side effects
*Painschab Malawi [17]	Cost-effectiveness Analysis	38 patients with DLBCL	Cost analysis	Compared to best supportive care, CHOP regimen is cost-effective ($189 incremental cost per DALYS averted) and within margin of willingness to pay. However, R-CHOP was borderline cost-effective (1204 incremental cost per DALYS averted)
*Ellis Malawi [18]	Interventional study	189 patients with Lymphoma 52% (62) HIV+ 13% had HL 41% DLBCL 11% low-grade NHL	Patient pathway-Treatment adherence	33% patients were not reachable for upfront mobile money transfers (MMT) 98% of those who received MMT came to clinic as per appointment MMT and upfront pay did not improve median treatment delay when compared to pre-intervention period
Adam Ethiopia [19]	Chart review	133 patients with HL (2014–19) 13% (12) were HIV+ 34.2% had stage 3 or 4 disease	Treatment outcome	Median age at diagnosis- 22 with no bimodal distribution and M:F ratio of 2.2:1 Mixed cellularity accounts for 50% of the HL diagnosed 82% treated with ABVD alone only 4.6% took chemo + radiotherapy 4-year OS was 83% with 100% survival for early stage disease
Fitzpatrick Kenya, Uganda, Botswana [20]	Cross-sectional study	32 patients with T-cell lymphoma 15% were HIV+	Clinicopathologic description	Median age of presentaion-45 M:F ratio = 1.7 41% of the cases were Primary T-cell lymphoma-Not Otherwise Specified 16% were EBV+
Jaquet Senegal,Cotedivore [21]	Case–control	117 NHL and 234 controls 7.7% HCV+, 12.8% HBV+ 14.5% HIV+ DLBCL-30% T-Cell NHL-30% 26.5% LG-NHL	Risk factors	Mature B cell account for 70% while T-cell lymphoma was 30% HIV was associated with increased NHL risk (OR = 3.3) Hepatitis B was associated with NHL risk (OR = 2.23) Hepatitis C (antibody) was associated with NHL risk (OR = 4.82) Only 28% of patients with HCV Ab had detectable HCV viral load
Polepole Zambia [22]	Chart review	240 cases of morphologically confirmed lymphomas HIV status- not available	Epidemiology	B-cell neoplasm was responsible for 92.5% NHL was responsible for 80% of the cases. DLBCL and BL were each responsible for 17.5% 16.3% were Classic HL with mixed cellularity and lymphocyte rich being most common subtypes 4.1% were unclassified
Wiggle South Africa [23]	Population- based cancer registry analysis	HIV associated lymphoma	Epidemiology	High-Grade-B cell lymphoma was the commonest HIV associated lymphoma at 70% followed by HL at 24% DLBCL is responsible for 45% of all HIV associated lymphoma
Chekol Ethiopia [24]	Case control	162 cases and 324 controls 2/3 of cases were above 45 years 2/3 of cases were illiterate 90% of cases live in rural area 43% are farmers 16% of cases HIV+ 9% of controls HIV+	Risk factor / patient pathway	Male sex (OR = 1.8), history of chemical exposure (OR = 11) and, lack of exercise (OR = 15) were associated with increased risk of NHL 70% of cases income less than 28 US dollars per month 35.8% and 16% of cases initially went to holy water and traditional healer, respectively before visiting the hospital
Antel South Africa [25]	Single arm interventional study	130 patients with lymphadenopathy 46% male, 49% HIV+, Median CD4-274, 76% were ECOG 0 or 1	Diagnostic and patient pathway	29% of patients with lymphadenopathy were diagnosed with lymphoma 22% of lymphoma patients were empirically treated for TB despite negative Gene-Xpert test Rapid access lymph node core biopsy clinic cut diagnostic interval from 48 days to 13.5 days
Okello Uganda [26]	Chart review	120 HIV−associated NHL	Treatment outcome	Patients on CHOP had a 1-year OS of 54.5% and those on DA-EPOCH had 80.2% Among DLBCL patients, a 1-year OS rate of 56.1% in the CHOP group and 100% in the DA-EPOCH group Adverse event reported—18% in the CHOP group and 25% in the DA-EPOCH group Completion of 6 or more cycles of chemotherapy was associated with improved OS
Cassim South Africa [27]	Chart review	181 DLBCL NOS cases 28% were HIV+ 51% were men Median age -52 and 39 (HIV +)	Treatment outcome HIV+ vs HIV−	Similar distribution of ABC /GCB subtypes by HIV status and age HIV+ with CD4 above 150 had similar survival compared with HIV− patients HIV+ patients with a CD4 < 150 had median survival less than 6 months DLBCL subtype (ABC/GCB), Ki-67 and age were not associated with differences in survival The 1-year, 2-year and 5-year OS were 65%, 52% and 40%, respectively
Tessa du Toit South Africa [28]	Chart review	609 patients who received biologicals 418 of 609 patients had rituximab exposure 261 had Hematologic malignancy	Treatment	Only 2% were on Isoniazid preventive treatment 3% of HM patients on rituximab developed TB TB incidence is twofold higher among patients treated with rituximab Risk of TB on rituximab is higher among HIV patients compared to HIV− (HR = 3) Median time to develop TB among patients on rituximab was 7 months
*Fedoriw Malawi [29]	Prospective cohort	59 cases of DLBCL were enrolled 54% were HIV+ median CD4 -117 and 60% on ART median age was 45 (HIV +) and 55 ( HIV−)	Treatment outcome HIV+ vs HIV−	OS and PFS was the same in HIV+ & HIV− groups No difference in genome expression profiling patterns between HIV+ and HIV− groups Ki-67 proliferation index ≥ 80% was associated with inferior OS in HIV+ DLBCL HIV status, expression cluster, and cell of origin classification were not associated with OS differences
Musekwa South Africa [30]	Chart review	68 Burkitt patients of which 50 adults Median age for adults was 37 78% of adults presented at stage3/4 92% of adult was HIV+ (CD4 < 200 in 53%)	Treatment outcome HIV+ vs HIV -	No difference in OS between different treatment groups (LMB-96 VS NON-LMP (Stanford, MACOP-B, Hyper-CVAD) Difference in OS between HIV+ (41%) and HIV− (57%) did not reach statistical significance 68% of adults with BL died at 2 years 39% of the deaths were prior to treatment Stage 1/2 had better OS (52%)
Vaughan South Africa [31]	Chart review	397 PBL patients in 2007- median age 38, median CD4 92 582 PBL patients in 2017 median age 40, median CD4- 146 95% of PBL patients were HIV+	Incidence, PRE/POST ART treatment outcome	PBL cases did not decrease despite improved ART coverage (5x) and viral suppression (6x) OS at 12 months (40%vs 41%) did not differ between 2007 & 2017 OS at 24 months (6% vs9%) did not differ between 2007 & 2017 Age, ART, AIDS, Viral suppression did not affect OS EBV negativity and LDH are markers of poor prognosis
Opie South Africa [32]	Chart review	49 HIV+ BL patients median age -37 61% had leukemic presentation 20% had complex karyotype Median CD4 count-240 cells	Treatment outcome clinical presentation	77% of the patients received high dose chemotherapy Median survival - 18 days without treatment and 267 days for those on high dose chemotherapy OS at 6 months and 12 months were 64% and 42%, respectively 5-year survival was 34% Age and CNS involvement were not associated with poor outcome
Parkin SSA [33]	Descriptive	GLOBOCAN 2018 cancer data for SSA	Risk factor	Population attributable fraction (PAF) of HCV on NHL was 0.9% for SSA EBV accounts for 81% of the continental BL cases and 75% of HL PAF of HIV for NHL patients was 12.7% The combined PAF for HIV, EBV, HCV and H. Pylori for NHL is 20% for males, 17% for females
Mezger SSA [34]	Chart review	A total of 506 patients from 11 cancer registries in 10 countries Patients with high-grade B cell NHL had a median age of 43 years, patients with low-grade B cell NHL and T cell NHL were aged 52 and 56 years, respectively	Diagnostics	Diagnosis was confirmed histologically in 76.2%, FNAC-only in 17.3% and without specimen analysis in 6.5% 57.9% of cases had no sub-classification 30.8% of the diagnosis did not include distinction between NHL and HL Among the subclassified, 55.8% had high-grade B cell, 29.5% low-grade B cell, 6.9% T-cell and 7.8% otherwise subclassified NHL DLBCL was the commonest (48%) followed by CLL/SLL and BL Only 6% of patients fulfilled the 5 clinical criteria laid out by NCCN harmonized guidelines for staging
Lorenzoni Mozambique [35]	Population- based cancer registry analysis	Maputo and Beira cancer registry	Incidence/ Epidemiology	NHL accounts for 4.2 and 5.8% of all cancers in males in Maputo and Beira, respectively NHL was responsible for 3% and 4.4% of all cancers among females in Maputo and Beira HL accounts for less than 1% of the cancer incidence in females and 1.6% in males
Manyau Zimbabwe [36]	Chart review	A total of 124 HIV patients were enrolled 27 patients were on R-CHOP while 70 patients were treated with CHOP median age of 42. 73% of patients were on ART	Treatment outcome HIV + NHL	Median OS was 11.2 months No difference in 12 months OS for CHOP group (44%) and R_CHOP group (50%) Male sex, age > 40, less than three cycles of chemotherapy and low SES are associated with poor prognosis 21% of patients have a treatment delay of more than 1 month after diagnosis
Zuze Malawi [37]	Prospective cohort and comparison with historical cohort	17 aggressive NHL patients (8 BL,8 PBL,1 PEL) Median age was 40. 65% HIV+ 41% had advanced disease	Treatment outcome EPOCH VS CHOP	EPOCH—59% achieved complete response 35% achieved partial response EPOCH- One‐year OS and PFS was 62% and 50%, respectively 29% died of disease progression while 18% died of treatment‐related mortality CHOP- 23% OS at 2 years for adolescents and adults
Griesel South Africa [38]	Laboratory- based Chart review	115 samples with both FNAC-FC and histology	Diagnostics FNAC-Flow cytometry VS histology	FNA-Flow cytometry can correctly predict B-cell NHL with an error rate of 10.41% High error rate for T-cell and HL All cases of HL were missed by the FNAC-FC
Bukirwa Uganda [39]	Population-based cancer registry analysis	Kampala cancer registry	Incidence/ epidemiology	Incidence (ASR/100,000) of NHL among males and females increased from 3.9 and 2.1 in 1991–1995 to its peak at 8.6 and 6.5 in the 2000–2006, respectively Incidence decreased to a level of 5.5 and 4.5 among males and females in the 2006–2011 Age-specific profile remains largely unchanged, with a peak in childhood (age group 5–9), followed by a progressive increase in incidence with age
Magangane South Africa [40]	Chart review	54 HIV+ and 205 HIV− DLBCL patients	Clinical presentation & Treatment outcome HIV+ vs HIV− DLBCL	HIV + DLBCL patients were younger at diagnosis compared to HIV− (40 vs 55) No difference in B symptoms, performance status and BM involvement complete cycles of chemo (5 +) were given in 56% of HIV+ and 66% of HIV− The 5-year OS was 56% versus 46% for HIV− versus HIV + DLBCL patients, respectively (p = 0.048)
Olson Tanzania [41]	Population- based cancer registry analysis	Bugando cancer registry	Epidemiology	Both HL and NHL are common in Males than females. NHL account for 73% of the lymphomas diagnosed NHL contributes to 4% of all cancers diagnosed while HL is responsible for 1% of the cancers 83.3% of HL and 80% of NHL in the registry were not classified to specific subtypes
De Boer Rwanda [42]	Historical cohort	85 HL patients—12%HIV+ ,56% advanced stage 70% with B symptoms	Treatment outcome (low dose vs high dose) cancer care delivery model (pathway)	74% of patients completed all 6 cycles 3-year overall survival -63% 3-year survival for low dose intensity was 36% v73% for high dose intensity Resource stratified protocols and implementation of quality indicators improves outcome in low resource settings
Leak Tanzania [43]	Descriptive study	209 cases of haematological malignancies NHL accounts for 44% of the cases	Cancer care delivery model	Adaptation and improvisation of existing infrastructure (Gene-Xpert machine and CD 4 count (flow cytometry) machine), local and international collaboration and teleconferencing helps close the gap in cancer care
Montgomery Malawi [44]	Prospective cohort	44 patients with DLBCL	Prognosis HIV+ vs. HIV−	High pre-treatment plasma EBV is associated with poor OS among HIV+ DLBCL but not HIV− patients (HR = 3.8) Median survival among EBV+ HIV+ patients was 16 days vs1534 days for EBV-HIV+ DLBCL
Koffi Cote d’ivoire [45]	Intervention study and cost analysis	NHL AND HL	Patient pathway—treatment cost	Financial difficulties are the main reason for treatment refusal and treatment abandonment Patient navigation system reduces treatment refusal and abandonment but not OS 58% discordance between morphology and IHC results were observed
*Painschab Malawi [46]	Prospective cohort	86 DLBCL patients treated with CHOP	Treatment outcome HIV+ vs HIV−	59% achieved complete response, 16% achieved partial response No difference in response rates b/n HIV+ and HIV− Median OS was 10.9 months and median PFS was 7.4 months One-year OS was 46% and two-year OS was 38% No difference in OS or PFS between HIV+ and HIV− groups
Iyer Botswana [47]	Cohort	109 NHL patients accounting for 12% and 3% of the cancers diagnosed among males and females in the cohort	Patient pathway—Male vs female	50% of NHL patients presented at stage 3/4 with no sex difference in staging at presentation Median time to treatment 7.9 months for males and 5.9 for females
Antel South Africa [48]	Prospective study	163 lymphoma patients (3/4 NHL and 1/4 HL) Median age 48 (38 for HIV +) 65% of patients came at stage 3/4	Patient pathway -diagnostics	10% of NHL patients and 27% of HL patients were on anti-TB 39% of patients had FNAC before biopsy Pathway analysis- patient interval is 4 weeks, health care practitioner interval is 7 weeks, referral and treatment interval are 2 weeks FNAC and late stage of presentation were significantly associated with delay 5-year OS was 46%, two-year OS was 52% HIV− patients had better OS for both NHL and HL
Feuchtner Ethiopia [49]	Retrospective population based cohort	40 NHL patients	Treatment, pathway	Only 40% of patients took greater than 85% of the chemotherapy recommended while in 30% of the cases chemo was either not planned or planned but not received
Dhokotera South Africa [50]	Chart review	NHL patients	Epidemiology	Cancer proportions were highest between the ages of 25–49 compared to age above 60 for HIV− groups Compared to HIV− patients, the odds of lymphoma subtypes in HIV patients shows Burkitt’s lymphoma (adjusted OR: 6.48), DLBCL (AOR 2.93 and, Diffuse immunoblastic large B-cell lymphoma (AOR 12.1)
Horner Malawi [51]	Linkage study	HIV patients on ART with median ART duration was 3.6 years	Epidemiology	Using 2001–2010 cohort, AIDS-defining cancers are responsible for 94% of malignancies among HIV+ patients on ART. NHL had an incidence rate of 11.7
Miguel Angola [52]	Population- based cancer registry analysis	HIV+ NHL patients	Epidemiology	NHL was the fourth commonest cancer and account for 4.5% of all cancers diagnosed It is responsible for 7.5% and 2.8% of cancers among males and females, respectively
Macharia Kenya [53]	Chart review	NHL patients	Epidemiology	30% of all cancers were attributable to infection in Kenya NHL was the fifth commonest cancer attributable to infection
Wawire Kenya [54]	Laboratory- based Chart review	90 DLBCL patients (54.5%) men and 75 women (45.5%); Median age at diagnosis was 50	Diagnostics/ epidemiology	40.6% were GCB-type and 59.4% were non–GCB-type DLBCL 10.9% has double expression of MYC and BCL2 Mean level of Ki67 expression was higher in the GCB group (36%) compared with the non-GCB group (26%) 9.7% were positive for EBV by EBER in situ hybridization, of which 75% were non-GCB
Swart South Africa [55]	Chart review	219 HL patients with 29% were HIV+ median age -32 no difference b/n HIV+ & HIV− and no bimodal distribution	Prognosis (HIV+ vs HIV -	HIV+ HL patients had higher risk of BM involvement (61% vs 28%) 33% of patients received empiric anti-TB prior to HL diagnosis (72% HIV+ HL patients vs.17% of HIV− HL) Common subtype among HIV− was NS-CHL while CHL unclassified in HIV+ HIV status (63% vs48%), BM involvement (70 vs40%), anti TB treatment, subtypes, LDH, CD4 impact the 5-year OS overall 5-year OS was 56% HIV+ patients with BM involvement had a 5-year OS of 18% vs48% in HIV− Lab proven TB was in 19% of HIV+ HL patients vs 6% among HIV− 31% HIV+ HL and 3% of HIV− HL didn’t receive chemotherapy due to clinical status
Egue Benin [56]	Population- based cancer registry analysis	119,086 cancer cases were registered from 2014–16	Epidemiology	ASR for lymphomas 2.2/10,000 among males and 1.1/10,000 among females. There was no categorization of HL and NHL HIV prevalence was 1.9%
Okongo Uganda [57]	Population- based cancer registry analysis	1627 cases of cancer from 2013–2016 Registry based in rural northern Uganda	Epidemiology	NHL (including unspecified lymphomas) were the most commonly diagnosed cancer in males (18.9%), with an ASR of 10.9 per 100,000 In females NHL was the second most common cancer (10.7%, ASR = 10.1) NHL incidence progressively increased with age among adults
*Painschab Malawi [58]	Prospective study	35 adolescents and adults with BL 15 were HIV+ Median age was 21 years 77%—stage III to IV disease	Treatment outcome HIV+ vs HIV−	HIV+ patients were older, had higher BMI, lower stage, and better ECOG and PS 50% achieved complete response,21% achieved a partial response, and 28% were refractory Median OS was 6.8 months while 1-year OS was 40% Patients with CR had 1-year OS of 70% Intensive chemotherapy (EPOCH & COPADM) was associated with decreased mortality vs CHOP HIV status and age didn’t affect OS
Rohner South Africa [59]	Cohort	210,898 adults from five continents were included 1,552 adults developed NHL after starting ART	Risk factor -HIV+ patients	NHL risk among HIV patients was similar in women and men No association b/n current CD4 cell count and NHL Risk of NHL was higher in SA women than in European women but similar among men Median time from ART start to NHL diagnosis was 1.1 years Median CD4 cell count at NHL diagnosis was 220 cells/µl median age at NHL diagnosis was 42.8 years NHL incidence rates were highest immediately after starting ART and decreased afterwards
Memirie Ethiopia [60]	Population- based cancer registry analysis	21,563 and 42,722 incident cancer cases in males and females, respectively	Epidemiology	ASR for NHL among males and females was 6.6/10,000 and 2.8/10,000, respectively For HL, ASR among males and females was 1/10,000 and 0.5/10,000, respectively
Naidoo South Africa [61]	Retrospective cohort	303 incident cases of HL (56.4% male and 43.6% female) 25% HIV+ HL patients	Epidemiology	Mean age at diagnosis was 33, with no difference based on HIV status. 55.8% HIV+ HL had a CD4+ count of < 350 cells/µl at the time of diagnosis, 39.0% had a count of < 200 cells/µl 23% had BM involvement 49.8% was CHL-NS while 23% was CHL-MC
Philips South Africa [62]	Chart review laboratory analysis	1215 BMB from patients with lymphoma (200HL&915NHL)	Epidemiology HIV+ vs HIV−	DLBCL and low-grade B-NHL are the commonest subtypes among HIV− NHL group In HIV+ NHL, BL was the commonest followed by DLBCL In HIV− HL, nodular sclerosis was the predominant subtype followed by unclassified CHL NS-unclassified is the commonest among HIV+ HL BM involvement at initial staging was 37.6% in NHL and 25.2% in HL Significant association between HIV+ status and BM involvement among BL but not DLBCL or PBL
Bigger Botswana [63]	Prospective study	104 newly diagnosed NHL patients.72% HIV positivity treatment given- CHOP (67.4%) or R-CHOP (32.6%) 58% of the total cohort had DLBCL	Treatment outcome HIV+ vs HIV -	HIV+ patients were younger (53 vs 39) & had aggressive disease (69% vs 84%) No association between HIV status and cancer stage or functional status 44.2% of patients died over a median follow-up of 11.9 months Disease progression was responsible for 89% of death while treatment toxicity was noted in 10.5% No association between treatment-related death and HIV status, stage at diagnosis, or PS One year OS-53%
Coghill Uganda [64]	Chart review	134 patients with NHL, and 63 patients with HL The median age of all patients was 43 years	Epidemiology HIV	71% of HL and 88% of NHL diagnosed at advanced stage 14.9% of NHL and 23.8% HL were diagnosed with TB HIV infection was significantly associated with the likelihood of presenting with advanced-stage disease
Inamasu South Africa [65]	Chart review	1880 hematologic malignancy patients aged 18—94 years	Epidemiology	NHL and HL accounts for 44% and 10% of hematologic cancers diagnosed 30–41 age group is the peak age for both HL and NHL DLBCL followed by BL and PBL were the commonest NHL subtypes
Menon Uganda [66]	Chart review	128 NHL patients median age-39.5 57% HIV+ ,15.6% TB	Treatment /pathway	90% presented with advanced disease Only 48% completed the recommended chemotherapy 25% died before completion of treatment while 47% were lost to follow up Low hemoglobin and advanced disease were predictors of mortality within 30 days
Mpunga Rwanda [67]	Case control	192cases of NHL and 61 cases of HL	Lymphoma/HIV	HIV increased the risk of both HL and NHL HIV was associated with DLBCL (OR = 6.6) and PBL (OR = 106), no association for T-cell NHL Nodular sclerosis CHL& mixed cellularity-CHL were the commonest HL HIV was significantly associated with lymphocyte-depleted CHL (OR = 20.) and mixed cellularity-CHL (OR = 12.0) and lymphocyte rich-HL (OR = 10.6)
Oelofse South Africa [68]	Population- based cancer registry analysis	2837 cases of lymphoid neoplasms diagnosed fb/n 2005–2013	Epidemiology	NHL had IR of 5.0 Per 100,000 for males and 3.9 per 100,000 for females HL had incidence rate of 0.6 and 0.4 Per 100,000 for males and females, respectively
Kaimila Malawi [69]	Prospective	72 HIV+ patients with lymphoma	Treatment outcome HIV+	NHL patients had OS of 48% and 38% at 12 and 24 months CHL patients had 100% OS at 12 and 24 months Lower CD4 and high viral load associated with bad outcome Chemotherapy together with ART results in long term CD4 recovery and viral suppression
Kaimila Malawi [70]	Prospective	21 relapsed or refractory: NHL (18) and HL (3) 62% were HIV+	Treatment outcome HIV+ vs HIV−	Median survival with salvage EPIC therapy without autologous HSCT was 4.5 months No difference in OS based on HIV status Majority of deaths are due to disease progression Disease progression was responsible for 91% of treatment discontinuation
Sissolak South Africa [71]	Chart review	35 patients with HIV+ BL or BL/DLBCL treated with LMB, HYPER-CVAD, Stanford or CHOP like regimen median age 38	Treatment outcome HIV+	2-year OS -38% and 23% event free survival No difference in OS between the two subtypes concomitant ART treatment is associated with OS (HR-0.19)
Westmoreland Malawi [72]	Prospective	31 CHL Patients Median age 19 33% were HIV+	Treatment outcome HIV+ vs HIV−	74% of patients has symptoms for > 6 months with over 50% presenting at stage 3 or 4 38% were empirically treated for TB Strong association between lymphoma and EBV: 75% EBER+ with 89% elevated viral load OS and PFS at 12 months -75% and 65%, respectively
Silas Nigeria [73]	Retrospective Cohort	32 HIV−and 8 HIV+ lymphoma patients NHL 87%	Treatment outcome HIV+ vs HIV−	HIV+ patients presented later than HIV− patients Mortality was associated with late-stage presentation but not HIV status Median survival for HIV+ patients was 2.1 years vs.7.6 years for HIV−(stage was a major determinant)
Buyego Uganda [74]	Retrospective Cohort	183 HIV associated lymphoma patients Median age was 35	Diagnosis misdiagnosis as TB	32% of patients were diagnosed as TB before lymphoma was confirmed 85% were identified as extrapulmonary TB Only 4/60 had bacteriologically confirmed TB Median time of treatment was 3.5 months before histology was done Late stage at presentation and chest pain are associated with TB misdiagnosis
Alli South Africa [75]	Chart review	504 head and neck lymphomas In 218 patients with known HIV status, 199 (91.3%) were HIV+ and 19 (8.7%) HIV−	Epidemiology	HL accounts for 10% of head and neck lymphomas while 90% are NHL 27.2% were nodal and 72.8% were extra nodal 99% of the extra nodal lymphomas were NHL PBL followed by DLBCL and BL were the commonest NHL subtypes
Chasimpha Malawi [76]	Population-based cancer registry analysis	population of Blantyre 2008–2010	Epidemiology	NHL accounts for 9.2% of the cancers diagnosed among males with ASR 10.3 per 100,000 Among females. NHL was the fifth commonest cancer accounting for 5.2% of the cases and ASR of 7.3/100,000
Perry Southern Africa [77]	Laboratory- based cross-sectional study	International NHL Classification Project	Diagnostics/ epidemiology	7.3% of cases were misclassified as NHL in southern Africa NHL patients in southern Africa are younger than EU and NA ( median age at diagnosis- 43 vs 60) higher relative proportion of high-grade lymphomas in Southern Africa when compared to EU and NA (51% vs 36%) low-grade NHL accounts for 36% compared to > 50% in NA and EU DLBCL was the most common subtype (38.2%) followed by FL (18.1%) and CLL/SLL (8.4%) Among the T-NHL, PTCL was the most common subtype (9.7%), followed by T-Lymphoblastic lymphoma (3.7%)
Ekanem Nigeria [78]	Population- based cancer registry analysis	719 cases of cancer were registered among Calabar residents from 2009–2013 HIV+ 4.4%	Epidemiology	HL was more common than NHL in the registry HL was the fourth commonest cancer accounting for 4.7%(ASR = 1.6/100,000) of all cancer diagnosis NHL account for 3.8%(ASR = 1.3/100,000) of all cancers in males HL and NHL account for 3.8% (ASR = 1.6/100,000) and 2.5% (ASR = 1.1/100,000) of all cancers diagnosed in females Comparison to population registries in Ibadan showed that, ASR for HL was 3–7 × higher in Calabar while the ASR for NHL was 5 times higher
Montgomery Malawi [79]	Lab-based cross-sectional study	85 pediatric and 82 adult patients Morphology vs Limited IHC plus morphology via weekly teleconference performed by local+ US pathologist Vs. US-based diagnosis via morphology+ IHC+ FISH	Diagnostics	5% treatment altering discordance between results from limited IHC vs US-based full diagnostics 8% treatment altering discordance between morphology Vs. US-based full diagnostics Morphology-only diagnosis was less specific when compared to morphology plus limited IHC with concordance rate of 76 Vs. 21% without affecting treatment decisions based on local guideline
Odutola Nigeria [80]	Population- based cancer registry analysis	Abuja and Enugu cancer registries 2012–2014	Epidemiology	23% of cancers in the registry were attributed to infectious causes with PAR of 44.4/100,000 ASR for NHL and HL in the registry 5/100,000 and 0.8/100,000, respectively ASR for NHL and HL attributable to EBV: 2.5/100,000 and 1/ 100,000, respectively
Morgan Malawi [81]	Chart review	328 cases of NHL diagnosed with H&E+ IHC	Diagnostics	Concordance rate of NHL diagnosis vis H&E only vs H&E+ IHC was 86% Diagnostic accuracy for 21-gene Nano String-based classifier was 96% for DLBCL and 88% for BL Nano String technology without expert pathology review can be feasible in SSA
Sall Senegal [82]	Chart review	40 cases of CLL diagnosed b/n 2011–2015 25 pts underwent FISH	Clinical and lab features	Mean age at diagnosis -61 Male to female ratio-3;1 82.5% had advanced disease at diagnosis-Binet stage-B/ C 70% of patients were CD-38 positive 68% had cytogenic abnormalities on FISH
Reddy South Africa [83]	Chart review	560 lymph node biopsy reports and 238 FNAC reports done prior to biopsy	Diagnostics	FNAC had good agreement with the biopsy for HL (Kappa = 0.774), and NHL (Kappa = 0.640) NHL and HL accounted for 15% and 8% of the lymph node pathology in HIV+ NHL and HL accounted for 9% and 8% of lymph node pathologies in HIV− HIV+ patients DLBCL accounted for 50% of NHL followed by BL at 17% In HIV− patients. DLBCL accounted for 21%
Onwubuya Nigeria [84]	Cross-sectional study	93 cases of lymphomas diagnosed based on morphology Male to female ration -1.9 Mean age -41.7	Epidemiology /diagnostics	5% of the cases were misclassified when IHC was performed 98.8% were B cell origin.80% were NHL while remaining are HL 53.8% of the lymphomas were high-grade DLBCL was the commonest NHL with 47% while all HL cases were classical HL BL was responsible for 8.75% of the lymphomas
Jaquet Benin, Côted'Ivoire, Nigeria and Togo [85]	Case-referent study	1,644 patients with a confirmed diagnosis of cancer and HIV serostatus	Epidemiology HIV+ vs HIV−	NHL accounts for 12% and 8% of malignancies among HIV+ patients and HIV− patients, respectively NHL was associated with HIV infection adjusted OR of 3.6 No significant associations between HIV infection and HL
Ndiaye Senegal [86]	Chart review	44 cases of NHL from 2005–2009 Mean age was 46 CLL and DLBCL accounted for 31% and 28%, respectively	Clinical features	Biopsy was performed in 52.3% of cases 70% had B symptoms and 37% were stage 4 at diagnosis CHOP was the commonest treatment protocol (36.3%) Rituximab was used in 2.3% Outcome: Regression of symptoms was observed in 61.3%, 11.4% were lost to follow and 27.4% had died High IPI patients had 100% death rate
Pather South Africa [87]	Lab-based cross-sectional study	52 DLBCL and 9 PBL cases and comparison based on HIV status	Diagnostic/ clinical features	HIV+ cases were younger at presentation and had extra nodal presentation. The OS of HIV+ DLBCL patients is worse than HIV− cases despite ART PBL has an overall poor outcome HIV+ DLBCL and PBL cases were associated with immunosuppression with mean CD count of 151 and 61, respectively BCL2 expression in germinal center associated with better survival
Mulwa-Babu Kenya [88]	cross-sectional study	49 cases of morphologically diagnosed CLL cases Median age 62	Diagnostic /clinical features	10.2% of cases had their diagnosis revised after IHC 28% had B symptoms 38.8% has stage 3 or 4 disease at presentation
Chokunonga Zimbabwe [89]	Population-based cancer registry analysis	28,319 cases of cancers among black population of Zimbabwe	Epidemiology	Trend analysis in incidence of lymphoma showed that ASR for NHL increased up to the year 2010 Stratification by age group showed that among the 15–39 age group, peak incidence was observed around the year 2001, and then a decline pattern was noted Among those older than 40, no decline in incidence was observed ASR for HL remained the same
Puvaneswaran South Africa [90]	Chart review	21 patients from rural KwaZulu-Natal	Diagnosis/pathway mis diagnosis as TB	85% of the patients diagnosed empirically with TB within one-year span of lymphoma diagnosis and were given a median of 5-month anti-TB therapy without clinical improvement
Bateganya Uganda [91]	Retrospective cohort	a total of 154 NHL patients 35% HIV+	Treatment outcome	73.8% patients received chemotherapy with 44% receiving more than 5 cycles No difference in chemotherapy rate based on HIV status HIV+ NHL patients on ART had comparable outcome with HIV− patients HIV+ NHL patients not receiving ART had increased mortality (HR = 8.99) compare to HIV− Presence of B symptoms was also associated with increased mortality (HR-2.09) 37.7% of the patients died in the first 12 months of follow-up
Olu-Eddo Nigeria [92]	Chart review	56 cases of HL	Epidemiology treatment outcome	Median age at diagnosis was 23 years with a bimodal peak in 11–15 and 21–25 age groups.66% presented at late stage. Mixed cellularity followed by lymphocyte depleted at 64.6 and 19.4 were the commonest subtypes. 5-year OS was > 80%
Abayomi South Africa [93]	Chart review	1076 cases of lymphoma 857 HIV− and 219 HIV+	Epidemiology HIV+ VS HIV−	Trend for increasing lymphoma incidence each year from 2002 to 2005 and stable in both the HIV+ and HIV− patients through to 2009 HIV−related lymphomas increased from 5% in 2002 to 37% in 2009 DLBCL (34%) followed by HL (20%) and follicular lymphoma (9%) were the commonest in the HIV−. Among the HIV+ patients, BL (31%) was the commonest followed by DLBCL (24%) and PBL (16%)

ABVD, Doxorubicin + Bleomycin + Vincristine + Dacarbazine, ART, antiretroviral treatment AOR, adjusted odds ratio, ASR, age-standardized rate, BL, Burkitt Lymphoma, BM, Bone marrow, BMI, Body mass index, CHL, classic Hodgkin lymphoma, CHOP, cyclophosphamide + vincristine + doxorubicin + prednisolone, CHOP-R, cyclophosphamide + vincristine + doxorubicin + prednisolone with Rituximab, CLL/SLL, chronic lymphocytic leukemia/small cell lymphocytic lymphoma, CNS, central nervous system, COPADM, Cyclophospahmide + vincristine + prednisolone + doxorubicin + Metotrexate, DLBCL, diffuse large B-cell lymphoma EBV, Epstein bar virus, EBER, Epstein bar virus encoded small RNAs, ECOG, Eastern cooperative oncology group, EPOCH, Etoposide + prednisolone + vincristine cyclophosphamide + doxorubicin, EU, European Union, FL, Follicular Lymphoma, FNAC Fine needle aspiration cytology FISH Florescence in situ hybridization GCP, germinal center B- cell, GLOBOCAN, Global Cancer Observatory, HBV, hepatitis-B virus, HCV, hepatitis-c virus,, H&E haematoxylin and Eosin HIV + human immune virus positive, HIV−, human immune virus negative, HL, Hodgkins lymphoma, HR, hazard ratio, HYPER CVAD, cyclophosphamide + vincristine sulphate + doxorubicin hydrochloride (Adriamycin) + and dexamethasone, H&E hematoxylin and eosin, HSCT, homologous stem cell transplant, IHC, Immunohistochemistry, IPI International prognostic index, LMP-MACOP NHL, Non Hodgkin lymphoma, NA, North America, OR, odds ratio, OS, overall survival, PS, performance status, PAR, population attributable ratio, PBL, plasmablastic lymphoma, PFS, progression free survival, PTCL, Primary T-cell Lymphoma, TB, tuberculosis SSA, sub-Saharan Africa

*Analysis was done on same cohort -Lymphoma cohort at Kamuzu Central Hospital

### Results

**Fig. 1:**
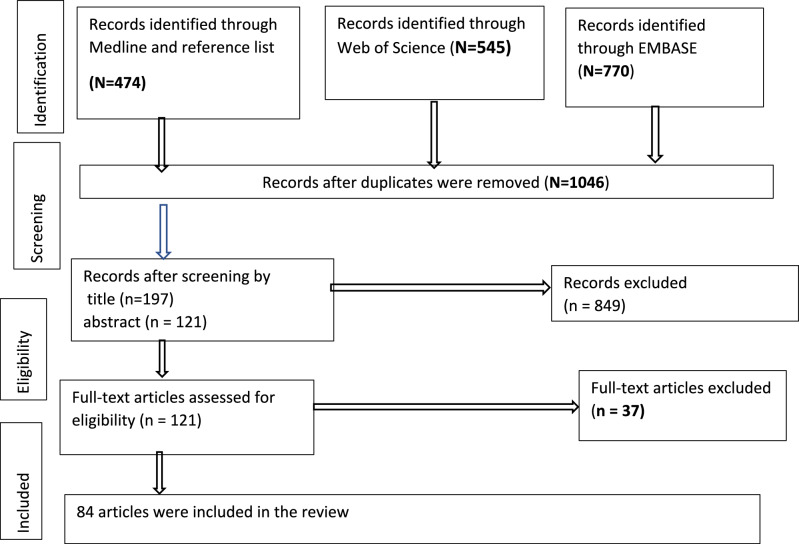
Preferred Reporting Items for Systematic Reviews and Meta-Analyses (PRISMA) flow diagram for study selection process

The database search identified a total of 1,789 articles from PubMed (Medline), Embase and Web of science. A total of 1,046 articles remained after duplicates were removed. Following title and abstract screening, 121 articles were eligible for full-text assessment. Among those, 37 articles were removed based on the exclusion criteria including conference proceedings (3 articles), case reports (7 articles) articles with pediatric lymphoma cases only (15 articles), sample size less than 15, (6 articles) Cutaneous lymphoma only (2 articles), articles with radiological lymphoma diagnosis (2 articles) and lastly, one migrant study to non-SSA countries leaving 84 to be included for the scoping review. The results of the search decision process are presented in the Prisma flow diagram in Fig. 1. Additionally, the findings of each study are charted in Table 1.

### Summary of findings

#### Study characteristics

**Fig. 2:**
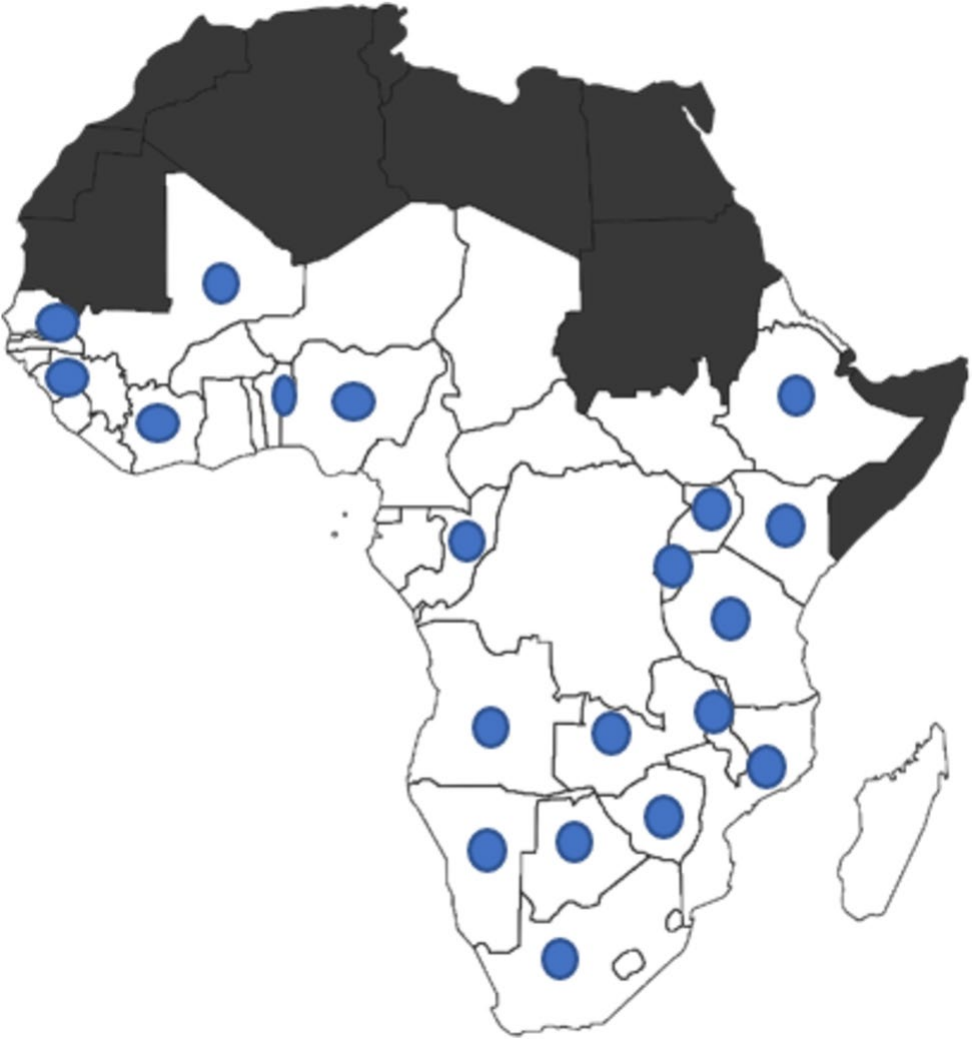
Map of Sub-Saharan African countries represented in the scoping review. Blue dots: Countries represented in the scoping review. Image copy-right CC0 1.0 Universal (CC0 1.0) Public Domain Dedication

The majority of the publications were from Southern Africa (49, 58.3%) with South Africa and Malawi contributing 24 and 16 articles, respectively and the remaining articles were from Botswana (3), Zimbabwe (3), Zambia (1), Mozambique (1) and Angola (1). One article was a multinational study from Zimbabwe and South Africa. The Eastern African sub-region had the next highest publication proportion; 21 articles, the most being from Uganda (8) followed by Kenya (5), Ethiopia (4), Tanzania (2), and Rwanda (2). There were 12 articles from the West African sub-region with seven of the articles from Nigeria, three articles from Senegal and one from Benin and a multinational study from Senegal and Cote d’Ivoire. Three articles were from the wider SSA region encompassing over 10 countries each. In total 20 countries from the SSA region were represented in this review. Figure 2 depicts countries represented in this scoping review.

The majority (83%) of the articles included in this scoping review were published in 2017 or after. A total of 17.86% (*n* = 15) articles were published in or after 2021 [10–24], while there were 23.8% (*n* = 20) publications from 2020 [25–44]. Analysis based on study design showed that 40 articles (47.6%) were chart reviews or laboratory-based cross-sectional studies^.^ [13, 19, 20, 22, 26–28, 30–34, 36, 38, 40, 43, 50, 51, 53–55, 62, 64–66, 71, 75, 77, 79, 81–84, 86–88, 90, 92, 93] Additionally, 15 (17.9%) articles were analysis based on cancer registries [14, 15, 23, 35, 39, 41, 52, 56, 57, 60, 68, 76, 78, 80, 89]. Meanwhile, 21(25%) articles employed a cohort study design. [10–12, 29, 37, 42, 44, 46–49, 58, 59, 61, 63, 69–74, 91] Case–control study design was used in four articles (4.7%) [21, 24, 67, 85].^.^ Finally, there were four interventional studies (4.7%) [16, 18, 25, 45] and one cost-effectiveness study [17].

### Incidence

A total of 14 population-based registries from Mozambique, South Africa, Uganda, Tanzania, Benin, The Gambia, Nigeria, Ethiopia, Malawi, Zimbabwe, and Angola assessed and reported age-standardized incidence rates(ASR) [14, 23, 35, 39, 41, 52, 56, 57, 60, 68, 76, 78, 80, 89]. One study assessed ASR for the whole SSA region [15]. NHL is by far the commonest lymphoma recorded across the registries with the exception of the Calabar registry which had a higher incidence of HL (2.3/100000) than NHL (1.3/10,000) [78]. The ASR for NHL ranges from 1.3 to 10.9 cases/100,000 [14, 23, 35, 39, 41, 52, 56, 57, 60, 68, 76, 78, 80, 89]. The lowest incidence of NHL was reported from Calabar registry in Nigeria while the highest incidence was from Gulu registry in Northern Uganda [57, 78]. Across all the registries the incidence of lymphoma was 1.5–2 times more common among males than females [14, 23, 35, 39, 41, 52, 56, 57, 60, 68, 76, 78, 89]. The examination of age-specific NHL incidence rates showed that, after a peak in the 5–15 age group (endemic Burkitt lymphoma), there was a progressive increase in the incidence with age [35, 39, 52, 56, 57, 60, 68, 76, 78].

The incidence of NHL in SSA has a positive association with the human immunodeficiency virus (HIV) prevalence in the region [21, 31, 39, 50, 51, 67, 85, 89, 93]. Overall HIV prevalence among NHL patients in the SSA region was 3.5–10 times higher than their overall national HIV prevalence [11, 16, 18, 19, 21, 22, 24, 27, 37, 58, 64, 66]. The lowest NHL incidence was reported in Calabar where the HIV prevalence was at 1.9% [78]. Overall, the southern Africa and Eastern Africa sub-regions, which had higher HIV prevalence, reported a higher NHL incidence compared to West Africa which has a relatively lower HIV prevalence [23, 35, 39, 41, 56, 57, 60, 78, 80, 85, 89, 93]. Three studies that looked at the trend in the incidence of NHL showed that the highest NHL incidence was observed in the early 2000s coinciding with the HIV pandemic and pre anti-retroviral treatment (ART) era in SSA [39, 89, 93]. A comparison analysis of NHL incidence in the Bulawayo cancer registry using the time periods 1963–72 and 2011–15 showed that there was 17 times and 6 times increment in the incidence of NHL among females and males, respectively [14]. According to two publications from Uganda and Zimbabwe, the incidence of NHL increased by 5–6.9% annually prior to 2006/7 [39, 89]. However, in the 2007–2015 period, annual reduction in the NHL incidence reaching up to a 10.5% was observed. Furthermore, the highest reduction in incidence was noted in the 15–45 age group [39, 89]. A recent global trend analysis from 1990 to 2019 also confirmed negative trends in incidence of NHL was noted in Zimbabwe, Ethiopia, South Sudan and Malawi [15].

The incidence of HL was also reported in seven population-based registries [35, 41, 60, 68, 78, 80, 89].^.^ The age-standardized ratio (ASR) for HL ranges from 0.4 to 2.3 cases/100,000 [35, 41, 60, 68, 78, 80, 89]. Sex distribution among HL patients across the region showed that HL is 1.6–2 times more common among males than females [19, 35, 60, 68, 78, 80, 89]. The highest incidence of HL was reported in Nigeria (1.6/100,000) [78]. The age specific incidence rate in HL in the SSA lacks a bimodal distribution with peak incidence in the 15–35 age group [19, 55, 60].

### Clinical and pathologic features

The median age of presentation for NHL patients in the region was between 43 and 50 years [20, 21, 25, 27, 29–31, 36, 37, 40, 54, 59, 62, 64, 74]. Late or advanced presentation is common in SSA with 60–88% of NHL patients presenting with stage-3 or stage-4 disease [10, 18, 25, 30, 40, 43, 47, 48, 64, 66, 82]. Over half of the patients report B symptoms [30, 40, 43, 47, 48, 64, 91]. HIV prevalence among NHL patients ranged from 60 to 73% in southern Africa to 12–37% in the Western Africa region [16, 18, 20, 21, 63, 66, 67, 73, 75, 78, 85, 91]. Tuberculosis (TB) is also a common co-morbidity among NHL patients with 10- 15% of NHL patients having confirmed TB [48, 64, 66, 74].

B-cell NHL was by far the commonest subtype accounting for 70–90% of diagnosed NHL [21–23, 34, 75, 77, 84]. Aggressive B-cell NHL accounts for over half of the NHL diagnosed in the region and two-third of the NHL diagnosed among HIV-positive patients [19, 22, 23, 56, 70, 72, 75, 76, 82]. Diffuse large B-cell lymphoma (DLBCL) is the commonest aggressive B-cell lymphoma accounting for 48–65% of the diagnosis [11, 22, 62, 77, 83, 84, 93]. In HIV-positive patients, Burkitt lymphoma (BL), DLBCL, and plasmablastic lymphomas are responsible for over three-fourth of all NHL [23, 62, 75, 77, 83, 84]. Low-grade B-cell lymphomas are uncommon and account for less than a third of the NHL diagnosed in the region [22, 62, 77]. Two studies that examined the distribution of T-cell lymphoma in the region showed that peripheral T-cell lymphoma was the most common subtype responsible for 41% of the T-cell NHL in the region [20, 77]. Furthermore, the prevalence of HIV among this group was assessed in one study at 15% [20]. A Malawian cohort that compared changes in NHL subtype distribution pre and post universal ART policy, and did not reveal a significant difference [11].

Studies from South Africa, Malawi and Botswana that compared clinical presentation between HIV-positive and HIV-negative DLBCL reported that, HIV-positive an HIV-negative patients showed similar clinical presentations including B symptoms, performance status and bone marrow involvement [29, 40, 44, 62, 63, 87]. However, HIV-positive DLBCL patients were younger, had aggressive disease and had a higher LDH compared to HIV negative patients [40, 87]. Similarly, HIV-associated BL patients experienced more extra-nodal involvement and leukemic presentations than HIV-negative patients [62].

As for HL, the mean age at presentation ranged from 16 to 22 in Eastern and West Africa sub regions [19, 92]. On the other hand, the median age at presentation in Southern Africa region ranged from 31 to 36 years [55, 61, 72]. While HIV prevalence among HL patients was 12–13% in the Eastern Africa region, its reported prevalence was 25–39% in the southern Africa sub-region [19, 42, 55, 61, 92]. Nodular sclerosis classic HL was the commonest subtype followed by mixed cellularity in Rwanda, Malawi and South Africa accounting for 49–53% of all HL [42, 55, 72]. In contrast, mixed cellular subtype was the commonest in Ethiopia and Nigeria at 50.4 and 64%, respectively [19, 92]. A third to half of the patients across the region present at advanced stage with 50–70% reporting B symptoms [19, 42, 55, 72, 92]. However, over 80% of patients had good performance status with ECOG 0-1 [19, 55].

### Risk factors

Infectious diseases such as Epstein Barr Virus (EBV), hepatitis C, and Helicobacter Pylori have also been associated with increased risk of NHL and HL in SSA [21, 33]. About 20–23% of NHL in the SSA region is attributable to these infectious agents with the population attributable fraction of HIV estimated at 12% [21, 33, 53, 85]. A multi-center case–control study from Senegal and Cote d’Ivoire showed that, hepatitis-B and hepatitis-C are associated with a two- and threefold increased risk of NHL, respectively [21]. Additionally, the study reported a threefold increased risk of NHL among HIV-positive patients [21, 85]. Among patients with HL, EBV was responsible for 71% of the HL cases reported [72]. A study from Ethiopia which evaluated non-infectious risk factors reported chemical exposures and lack of exercise as major risk factors for NHL [24].

### Diagnostics

Diagnosis of lymphoma in SSA is often delayed, gets frequently misdiagnosed as TB and also lacks sub-classification [25, 34, 45, 48, 74, 77, 83, 90]. Over two-thirds of the NHL diagnosis in the SSA region are obtained via histology-only, while over a fifth of patients are diagnosed either by fine needle aspiration cytology (FNAC)-only or on clinical grounds alone [34]. In an analysis across 11 cancer registries among of NHL patients in SSA, diagnoses were confirmed histologically in 76.2%, with FNAC-only in 17.3% and clinically without specimen analysis in 6.5% [34]. Furthermore, in one study, 39% of lymphoma patients had FNAC prior to biopsy causing a delay in diagnosis [48]. Two studies which assessed diagnostic accuracy of FNAC against biopsy showed conflicting results [38, 83]. In one study from South Africa, FNAC performed very well with only 11% error rate when compared with immunohistochemistry (IHC) in diagnosing B-cell NHL but an unacceptably high error rate for HL and T-cell lymphomas [38]. Another study, also from South Africa, reported a 66% and 77% agreement between FNAC and biopsy among NHL and HL patients, respectively [83].

Morphology is the mainstay of diagnosis across SSA, with few studies reporting IHC results [34, 45, 77, 81, 84]. Misclassification among lymphoma patients in SSA is significantly higher than the global North [77]. Furthermore, histologically diagnosed cases were subclassified in only 50.8% while cytologically diagnosed cases were subtyped in 37.3% [34]. In 57.9% of the cases, no sub-classification was identified of which 69.2% were unclassified NHL, while in 30.8%, there was no distinction between NHL and HL [34]. A report for the NHL classification project reported a 7.3% discordance between the initially reported diagnosis and a second review by the international Lymphoma consortium group while a cross-sectional study from Nigeria showed a 5% discordance between morphology and IHC review [84]. Similarly, studies from Cote d’Ivoire, Malawi and Kenya that compared IHC to morphologic examination reported 58.8%, 14% and 10.2% diagnostic discordance, respectively [45, 81, 88]. Interventions such as limited IHC plus teleconferencing with a US pathologist to perform sub- classification showed encouraging results with less than 8% major discordance, however, morphology only was less specific with concordance rate of less than 21% for subclassifying subtypes [79]. Another innovative diagnostic modality that employs Nanostring technology without expert pathologist in Malawi showed promising results with a diagnostic accuracy of 96% and 88% among DLBCL and BL patients, respectively [81].

### Treatment and treatment outcomes

Cyclophosphamide, doxorubicin, vincristine and prednisolone (CHOP) chemotherapy regimen was the commonest treatment regimen prescribed for NHL in the region with a few centers providing rituximab plus CHOP for selected patients [26, 27, 36, 37, 46, 63, 71, 86]. One-year overall survival ranged from 45 to 55% among patients receiving the CHOP regimen [26, 27, 30, 40, 46, 48, 63, 69]. Comparable treatment outcome was observed between HIV-positive patients on ART and HIV-negative NHL patient. One single-arm interventional study among NHL patient treated with R-CHOP showed a 68% overall survival and 11% treatment-related mortality [16]. Active TB infection is shown to be a major complication among patients on rituximab [28]. According to a study from South Africa, the incidence of TB doubled among patients taking Rituximab [28]. An even higher incidence (threefold) of tuberculosis was observed among HIV-positive patients [28].

A few studies that assessed feasibility of an intensive chemotherapy protocol that included the dose adjusted Etoposide, prednisolone, doxorubicin, cyclophosphamide and vincristine (DA-EPOCH) showed a good response with 62–80% one-year overall survival and 18–25% treatment-related adverse events [26, 27, 37, 58]. Treatment outcome is comparable among HIV-positive NHL patients on ART and HIV-negative patients [16, 27, 29, 30, 46, 63, 69, 73, 91]. However, HIV patients not on ART had poor overall survival at 18% [91]. One study that looked into PBL however showed that viral suppression or ART did not improve overall survival [31].

The majority of HL patients in SSA are managed with chemotherapy regimen-Doxorubicin, Bleomycin Vincristine and Dacarbazine (ABVD) without radiotherapy with the exception of South Africa [19, 42, 55, 72]. Four studies that evaluated treatment outcome among HL patients showed a one-year overall response rate approaching 75–85% and even higher response rate was observed among patients with early stage disease [19, 42, 55, 72].

### Prognostic factors

Studies that evaluated prognostic factors among NHL patients showed that, advanced stage, bone marrow involvement, not being on ART, less than five cycles of chemotherapy, empirical TB treatment were associated with poor prognosis [26, 28, 30, 35, 65, 90]. Among the NHL subtypes, PBL and BL patients had a worse prognosis than DLBCL patients [29, 30, 63, 70]. In contrast, age, HIV status, viral suppression, classification based on cell of origin (GCB vs non-GCB) were not associated with outcome [25, 26, 45, 62].

### Patient pathway

Lymphoma patients in SSA have delayed presentation and long diagnostic intervals [24, 47, 48, 66]. A study from Botswana reported 7.9-month median time to treatment [47]. Similarly, a study from Ethiopia reported that a third of NHL patients seek help from traditional healers before visiting hospitals [24]. While patient intervals (time elapsed before health care contact) are responsible for late-stage presentation, diagnostic delay was also a common observation in the region. Two studies from South Africa and Cote d'Ivoire, that explored pathways to care identified that excess reliance on FNAC and difficulty of accessing lymph node biopsy were associated with diagnostic delay [45, 48]. Implementation of rapid access core lymph node biopsy and online referral system in South Africa has reduced the diagnostic interval from 6 to 2 weeks [25].

Misdiagnosis and empirical TB treatment was also common across the region [30, 48, 55, 74, 90]. According to a study from rural South Africa, 85% of lymphoma patients were empirically treated for tuberculosis within a year of their lymphoma diagnosis [90]. Another hospital-based study from South Africa also showed that 10 and 27% of NHL and HL patients respectively were on TB treatment [48]. In the Malawi cohort, 38% had received empiric tuberculosis treatment for lymphadenopathy [72]. Similarly, in the South African retrospective cohort study, 72% of HIV-positive HL patients received TB therapy even though only 19% has lab proven TB [55]. A study from Uganda also showed that 32% of NHL patients were empirically treated for tuberculosis while biologically confirmed TB was only evidence in four out of 60 cases [74].

Treatment delay, refusal or treatment abandonment was common, ranging from 37 to 60% in some centers [24, 36, 45, 48, 49, 66]. In contrast, some centers in Southern Africa have over 90% treatment completion rates [46]. Lack of financial support, family opposition, interference with traditional medicine, transportation obstacles, discouragement were identified as factors associated with treatment abandonment [24, 45, 66]. A study from Ethiopia also showed that only 40% of patients took more than 85% of the prescribed chemotherapy while in 30% of NHL patients either chemotherapy was not planned or patient did not receive [49]. One study that evaluated a visit reminder and mobile money transfer intervention to improve treatment delay in Malawi did not show benefit in terms of treatment delay [18]. Furthermore, Ambulatory medical assistance in Côte d'Ivoire showed impact on treatment abandonment and refusal but not overall survival [45].

Finally, cost-of-care analysis from a Malawian cohort showed that treatment costs are almost fivefold annual gross national income per capita [17]. In Cote d'Ivoire, the cost of each cycle of CHOP regimen was estimated at 360 USD while 40% of the patients had a monthly income of less than 100 USD [45]. A recent cost-effectiveness comparison between CHOP and R-CHOP in Malawi showed that generic CHOP is cost-effective and well under the willingness to pay margin while R-CHOP was likely cost-effective as the incremental cost-effectiveness ratio was very close to the WHO willingness to pay threshold. [17]

### Cancer care models

Two studies evaluated models of cancer care in resource limited settings [42, 43]. A study from Rwanda that employed task shifting of cancer care to Internists and pediatricians, resource-adapted treatment protocol, strict adherence to protocol, teleconferencing and patient tracking measures showed promising results [42]. Similarly in Tanzania, task shifting, improvising CD4 machine and GeneXpert machine for flow cytometry tests as well as molecular diagnostics has dramatically improved quality of care [43].

## Discussion

National or regional cancer control plans (NCCP) backed by robust evidence and policy are vital in addressing the global cancer burden as well as improving treatment outcome among patients. [94] Lack of operational and evidence-based NCCP have significantly contributed to disparities in cancer control and outcome globally. [95] According to the International Cancer Control Partnership (ICCP) portal (https://www.iccp-portal.org/map), 24 SSA countries have NCCP. [96] However, as per the consensus by the Africa Cancer Research and Control ECHO Program, designing or implementing a fully operational NCCP has been a major challenge in the region. [95] Our scoping review attempted to contribute to this knowledge gap by summarizing the existing evidence on the epidemiology, clinical characteristics, and therapeutic challenges of lymphoma patients in the SSA region with the aim of facilitating evidence-based policy decisions in lymphoma care and control in the region.

To our knowledge, this is the first scoping review to report on the existing published literature on lymphoma In SSA region. Overall, this scoping review has identified the following key findings. Over 80% of the available articles were published in the last five years, demonstrating an increasing trend in publications from the region. However, over half of the published literatures came from three countries namely, South Africa, Malawi and Uganda highlighting a major publication gap from other sub-regions and individual countries notably in highly populated countries like Nigeria and Ethiopia.

Our scoping review showed that the reported incidence (ASR) rates from the cancer registries in SSA are lower than the GLOBOCAN estimates for the global north as well as Asia and the Caribbean. However, we should be cautious in interpreting such reports as the majority of cancer registries in SSA with few exceptions are sub-national and urban oriented representing less than 10% of the population. In addition, further caution is warranted in interpreting ASR from cancer registries in SSA as likely underestimation due to limited diagnostic capacity, less than efficient cancer care pathways and barriers to health seeking behavior. Reported rates can only capture patients in the formal health system leaving out those who opt for traditional medicine (which is not uncommon in the region), as well as those who have not sought any care.

Furthermore, the reported median age at diagnosis in the SSA was younger than other published reports globally. However, this finding should also be carefully interpreted in light of the age structure of the region whereby only 3% of the population is above the age of 65 years.

We have also noted multiple studies that showed a higher proportion of aggressive B-cell NHL among the reported cases and one of the lowest prevalences of indolent NHL such as follicular lymphomas worldwide. While further studies are required to understand whether this observation is an inherent characteristic of the population, multi layered factors such as HIV infection, lack of diagnostics and poor health access and awareness of potential lymphoma related symptoms, particularly for indolent lymphomas could provide partial explanations for this discrepancy. While SEER data from the US reported differential incidence rates of lymphomas by race, with a 19–64% lower incidence of lymphomas and three times lower incidence of follicular lymphoma among blacks than non-Hispanic whites, suggesting a genetic component, applying such data to the SSA region may not be appropriate and requires further study^.^[97].

This review, by virtue of its dependence on published material, was not able to assess the true burden of lymphoma, including the population who did not present to health facilities, as well as those who sought care but were misdiagnosed or undiagnosed with lymphoma. This highlights the need for community-based pathway studies to estimate undiagnosed cases. Focusing on those who presented to health facility, we note existing gaps in the diagnosis and treatment aspect of lymphoma. Our review identified that diagnosis of lymphoma in SSA is often delayed, lacks subtype classification with over 20% patients being diagnosed either clinically or via FNAC. Heavy reliance on FNAC, and morphology-based diagnosis without IHC confirmation is the main practice across the region. In addition, overlapping clinical presentations between lymphoma and extra-pulmonary tuberculosis, the high TB burden in the region, difficulties in accessing lymph node biopsies and poor pathology infrastructures were noted. These contributed to (unacceptably) high rates of presumptive TB diagnoses and treatment among lymphoma patients, with 30–85% clinically presumed to have TB and unsuccessfully treated for the infection before a histological diagnosis of lymphoma was actually made, resulting in significant treatment delay. Given the challenges of accessing health care and patient pathways and poor overall patient follow up, these figures could just represent the “tip of the iceberg” with a significant number of lymphoma patients being lost from care without being diagnosed.

For those who do seek care, the lack of reliable and standardized diagnosis has also limited the potential for optimal therapies as recommended in the resource- stratified guidelines for SSA [98].Such treatment options such as Rituximab, could potentially be associated with a 10–15% improvement in overall survival and event- free survival of lymphoma patients were proven to be cost-effective in the Malawian interventional study^.^ [17, 99]. However, encouraging trends such as tele-pathology, introduction of IHC, and adaptation of flow cytometry machines beyond their use for CD4 counts to immunophenotyping lymphoma were shown to improve diagnostic accuracy in some regions. In addition, a recent interventional study from South Africa published outside of our scoping review eligibility period showed that physician led biopsy clinics that combine GeneXpert with core needle biopsy correctly identified 96% of lymphomas thereby providing a cost -effective yet reliable diagnostic pathway for the SSA region. In addition, 27% of lymphadenopathies were confirmed to be lymphoma while 33% were TB with no overlap between the two diagnose [100]. These findings calls to question the current differential diagnosis and treatment pathway that presumes that patients who present with lymphadenopathy are suffering from tuberculosis. In addition, it demonstrates that when rapid and accurate diagnostic techniques are applied, misclassification of lymphoma as tuberculosis can be totally abrogated, leading to appropriate treatment. Finally, these findings underscore a need for awareness of lymphoma signs and symptoms, both among patients and care-givers, as well as redesigning clinical pathways and algorithms that also emphasize on lymphoma.

Finally, we identified that, while NHL is a treatable and potentially curable disease in the Western Hemisphere, one year survival across the region remains around 50%, much lower than the 80–90% overall survival in Europe and North America. Interestingly, our findings showed that despite HIV being a major risk factor in the region, treatment outcome was comparable among HIV-positive NHL patients on ART and HIV-negative patients. Despite the presence of few interventional studies, existing knowledge shows that treatment is well tolerated with fewer adverse effects. Poor outcome was mainly attributed to challenges in patient pathways such as diagnostic delay, late presentation, misdiagnosis, lack of medications and treatment abandonment. Furthermore, non-clinical aspects of cancer care such as lack of financial support, family opposition, interference with traditional medicine, transportation obstacles have major role in dictating health seeking behavior, treatment follow up and completion of care in the region. treatments costs in the region were over the willingness to pay margin for the majority of the population.

Our review has also identified several gaps in the existing literature. Published reports on those who do not seek care, or those who were missed by the system due to misdiagnosis were not available. Secondly, publications assessing the epidemiology of low-grade lymphomas are very scarce in the region. Additionally, the role of non-infectious risk factors for lymphoma in SSA remains unexplored. Moreover, 48% of the data sources were derived from chart reviews and retrospective analysis. While chart reviews can offer valuable insights, they often come with inherent limitations related to data quality and representativeness. In addition, the sample sizes of most studies included in the review were relatively small, which raises concerns about the generalizability of the findings. Additionally, the cancer registries utilized in this review were primarily urban-oriented and were hampered by issues such as data quality, diagnostic misclassification, and incompleteness. These shortcomings may have compromised the accuracy of the reported incidence rates and may have introduced biases in the findings.

Thirdly, only few studies and cancer registries used the WHO classification for lymphoma histology, thereby, limiting the quality of the existing data and also hampering further comparisons with other studies and regions. Finally, interventional and well-designed prospective cohort studies are lacking with the exception of the Malawian cohort, hence limiting potential systematic review on role of HIV infections, treatment outcome or prognosis.

### Study limitations

We were only able to include data from published articles. The grey literature was not assessed. However, we have extensively looked for published data from cancer registries to provide a comprehensive summary. Secondly, multiple articles specifically from Malawi, South Africa and Nigeria were published using the same patient groups. We have made attempts to only include those with a different research question.

Finally, although scoping review guidelines by Arksey and O’Malley suggests consultations with key stakeholders, we did not include a consultation process as such consultations on lymphoma already exist in the literature [101].

## Conclusion and recommendation

A lymphoma care model that addresses the major challenges summarized in our findings—such as delayed presentation, diagnostic delay, misdiagnosis, treatment abandonment, and poor diagnostic capacity—is vital for improving patient outcomes in the region. In settings where basic access to care and diagnostic accuracy are limited, it is essential to focus on innovative interventions to strengthen these foundational elements. Additionally, the region's unique epidemiological challenges, including high HIV and TB prevalence, necessitate an integrated, multidisciplinary approach to patient care, where the possibility of lymphoma, a highly treatable disease, is not ignored. While the successes of HIV care in SSA, such as task shifting, offer valuable insights, these strategies must be adapted carefully to ensure that diseases like lymphoma are considered in the differential diagnosis by devising accurate diagnostic algorithms. Improvement in all of these aspects of care, and high-quality research will promote equity for lymphoma patients in SSA.

## Data availability statement

The data underlying this scoping review are available within the article and in the appendix. All data used in this review were extracted from publicly available literature, and no new primary data were collected. The list of articles included in the scoping review, along with the extraction template is available in the review itself. For additional information, please contact the corresponding author.

## Electronic supplementary material

Below is the link to the electronic supplementary material.

Supplementary file1 (DOCX 108 KB)

## Appendix 1: Search strategy

1. EMBASE

**Table:**

#	Query	Results
12	#10 AND #11	770
11	'adult'/exp OR 'adult':ti,ab,kw OR 'adults':ti,ab,kw OR 'grown-ups':ti,ab,kw OR 'grownup':ti,ab,kw OR 'grownups':ti,ab,kw OR 'adolescent'/exp OR 'adolescent':ti,ab,kw OR 'teenager':ti,ab,kw	12190809
10	#9 AND ('Article'/it OR 'Article in Press'/it OR 'Conference Paper'/it OR 'Conference Review'/it OR 'Preprint'/it OR 'Review'/it OR 'Short Survey'/it)	1144
8	#4 AND #7 AND [2012–2022]/py	2173
8	#4 AND #7	4467
7	#5 OR #6	882589
6	'lymphoma'/exp	375261
5	lymph*:ti,ab,kw AND (tumor*:ti,ab,kw OR tumor*:ti,ab,kw OR malignan*:ti,ab,kw OR neoplasm*:ti,ab,kw OR cancer*:ti,ab,kw)	662971
4	#1 OR #2 OR #3	523207
3	'africa south of the sahara'/exp	312040
2	angola:ti,ab,kw OR benin:ti,ab,kw OR botswana:ti,ab,kw OR 'burkina faso':ti,ab,kw OR burundi:ti,ab,kw OR cameroon:ti,ab,kw OR 'cape verde':ti,ab,kw OR 'central africa':ti,ab,kw OR 'central african republic':ti,ab,kw OR chad:ti,ab,kw OR comoros:ti,ab,kw OR congo:ti,ab,kw OR 'ivory coast':ti,ab,kw OR djibouti:ti,ab,kw OR eritrea:ti,ab,kw OR eswatini:ti,ab,kw OR ethiopia:ti,ab,kw OR gabon:ti,ab,kw OR gambia:ti,ab,kw OR ghana:ti,ab,kw OR guinea:ti,ab,kw OR kenya:ti,ab,kw OR lesotho:ti,ab,kw OR liberia:ti,ab,kw OR madagascar:ti,ab,kw OR malawi:ti,ab,kw OR mali:ti,ab,kw OR mayotte:ti,ab,kw OR mozambique:ti,ab,kw OR namibia:ti,ab,kw OR niger:ti,ab,kw OR nigeria:ti,ab,kw OR rwanda:ti,ab,kw OR sahel:ti,ab,kw OR senegal:ti,ab,kw OR 'sierra leone':ti,ab,kw OR somalia:ti,ab,kw OR 'south africa':ti,ab,kw OR sudan:ti,ab,kw OR tanzania:ti,ab,kw OR togo:ti,ab,kw OR uganda:ti,ab,kw OR zambia:ti,ab,kw OR zimbabwe:ti,ab,kw	446167
1	'subsaharan africa':ti,ab,kw OR 'sub-saharan africa':ti,ab,kw OR 'black africa':ti,ab,kw OR 'africa south of the sahara':ti,ab,kw	36700

2. PUBMED

**Table:**

#	Query	Results
15	#11 AND #14	474
14	#12 OR #13	9348306
13	adult*[Title/Abstract] OR adoles*[Title/Abstract] OR teenager*[Title/Abstract]	1824280
12	("Adult"[Mesh]) OR "Adolescent"[Mesh]	8600876
11	#9 NOT #10	853
10	("Letter" [Publication Type]) OR "Editorial" [Publication Type]	1861281
9	#4 AND #7	872
8	#4 AND #7	2894
7	#5 OR #6	538763
6	"Lymphoma"[Mesh]	186333
5	(Lymph*[Title/Abstract]) AND (tumor[Title/Abstract] OR tumor[Title/Abstract] OR malignan*[Title/Abstract] OR neoplasm*[Title/Abstract] OR cancer*[Title/Abstract])	418913
4	#1 OR #2 OR #3	469763
3	"Africa South of the Sahara"[Mesh]	256573
2	Angola[Title/Abstract] OR Benin[Title/Abstract] OR Botswana[Title/Abstract] OR "Burkina Faso"[Title/Abstract] OR Burundi[Title/Abstract] OR Cameroon[Title/Abstract] OR "Cape Verde"[Title/Abstract] OR "Central Africa"[Title/Abstract] OR "Central African Republic"[Title/Abstract] OR Chad[Title/Abstract] OR Comoros[Title/Abstract] OR Congo[Title/Abstract] OR "ivory coast"[Title/Abstract] OR Djibouti[Title/Abstract] OR Eritrea[Title/Abstract] OR Eswatini[Title/Abstract] OR Ethiopia[Title/Abstract] OR Gabon[Title/Abstract] OR Gambia[Title/Abstract] OR Ghana[Title/Abstract] OR Guinea[Title/Abstract] OR Kenya[Title/Abstract] OR Lesotho[Title/Abstract] OR Liberia[Title/Abstract] OR Madagascar[Title/Abstract] OR Malawi[Title/Abstract] OR Mali[Title/Abstract] OR Mayotte[Title/Abstract] OR Mozambique[Title/Abstract] OR Namibia[Title/Abstract] OR Niger[Title/Abstract] OR Nigeria[Title/Abstract] OR Rwanda[Title/Abstract] OR Sahel[Title/Abstract] OR Senegal[Title/Abstract] OR Sierra Leone[Title/Abstract] OR Somalia[Title/Abstract] OR "South Africa"[Title/Abstract] OR Sudan[Title/Abstract] OR Tanzania[Title/Abstract] OR Togo[Title/Abstract] OR Uganda[Title/Abstract] OR Zambia[Title/Abstract] OR Zimbabwe[Title/Abstract]	386601
1	"subsaharan Africa"[Title/Abstract] OR "sub-saharan Africa"[Title/Abstract] OR "black Africa"[Title/Abstract] OR "Africa south of the Sahara"[Title/Abstract]	37305

3. WOS

**Table:**

#	Search query	Results
5	#1 AND #2 and 2022 or 2021 or 2020 or 2019 or 2018 or 2017 or 2016 or 2015 or 2014 or 2013 or 2012 (Publication Years) and Article or Review Article (Document Types)	545
4	#1 AND #2 and 2022 or 2021 or 2020 or 2019 or 2018 or 2017 or 2016 or 2015 or 2014 or 2013 or 2012 (Publication Years)	567
3	#1 AND #2	1079
2	Lymph* AND (tumor OR tumor OR malignan* OR neoplasm* OR cancer*) (Title) OR Lymph* AND (tumor OR tumor OR malignan* OR neoplasm* OR cancer*) (Abstract)	357211
1	"sub-saharan Africa" OR "sub-saharan Africa" OR "black Africa" OR "Africa south of the Sahara" OR Angola OR Benin OR Botswana OR "Burkina Faso" OR Burundi OR Cameroon OR "Cape Verde" OR "Central Africa" OR "Central African Republic" OR Chad OR Comoros OR Congo OR "ivory coast" OR Djibouti OR Eritrea OR Eswatini OR Ethiopia OR Gabon OR Gambia OR Ghana OR Guinea OR Kenya OR Lesotho OR Liberia OR Madagascar OR Malawi OR Mali OR Mayotte OR Mozambique OR Namibia OR Niger OR Nigeria OR Rwanda OR Sahel OR Senegal OR Sierra Leone OR Somalia OR "South Africa" OR Sudan OR Tanzania OR Togo OR Uganda OR Zambia OR Zimbabwe (Title) OR "subsaharan Africa" OR "sub-saharan Africa" OR "black Africa" OR "Africa south of the Sahara" OR Angola OR Benin OR Botswana OR "Burkina Faso" OR Burundi OR Cameroon OR "Cape Verde" OR "Central Africa" OR "Central African Republic" OR Chad OR Comoros OR Congo OR "ivory coast" OR Djibouti OR Eritrea OR Eswatini OR Ethiopia OR Gabon OR Gambia OR Ghana OR Guinea OR Kenya OR Lesotho OR Liberia OR Madagascar OR Malawi OR Mali OR Mayotte OR Mozambique OR Namibia OR Niger OR Nigeria OR Rwanda OR Sahel OR Senegal OR Sierra Leone OR Somalia OR "South Africa" OR Sudan OR Tanzania OR Togo OR Uganda OR Zambia OR Zimbabwe (Abstract)	679128
